# Numerical Study of the Optimum Fiber Content of Sealing Grease Using Discrete Element Method

**DOI:** 10.3390/ma15103485

**Published:** 2022-05-12

**Authors:** Xiong Zhou, Yingjie Wei, Yuyou Yang, Pengfei Xu

**Affiliations:** School of Engineering and Technology, China University of Geosciences (Beijing), Beijing 100083, China; zhou_xiong@cugb.edu.cn (X.Z.); yangyuyou@cugb.edu.cn (Y.Y.); xupengfei@cugb.edu.cn (P.X.)

**Keywords:** fiber-reinforced composites, fiber content, subway tunnel, fiber aspect ratio, numerical simulation

## Abstract

A sealing grease plays a crucial role in the sealing of shield tails. Its pumpability and pressure sealing resistant sealing performance are greatly affected by the fiber content. In this study, discrete element method models were used to simulate the pressure-resistant tests of sealing grease in order to investigate the influence of viscosity grade and fiber’s aspect ratio on the optimum fiber content of sealing grease. Meanwhile, the rationality of the optimum fiber number determined based on the sealing performance was verified with the unbalanced force and fiber area proportion obtained in the simulation, of which the variation curves with the increasing fiber number were practically identical. The simulation results elucidated that the viscosity of grease had little effect on the optimum fiber content for sealing grease. However, the increase in viscosity can improve the sealing effect, and increasing the fiber’s aspect ratio can reduce the fiber number to reach a specific seal state. Based on the analysis of the total number of fiber spheres for the models with different fiber’s respect ratios, it can be concluded that the sealing grease sample made of the same fiber material and quality can reach the same seal state and seal effect, independent on fiber’s aspect ratio.

## 1. Introduction

Sealing grease for the tunnel boring machine consists of fiber, anchoring agent, powder, and base oil. They are working together to ensure that there is no gap between the shield tail and lining [1]. Fiber, including polypropylene fiber, plant fiber, and cotton fiber, plays a skeleton role in sealing grease and has an important effect on the high-pressure sealing performance. Anchoring agent, such as polyisobutylene and resin, mainly affects the adhesive ability between fiber and matrix, which can improve the adhesion, stickiness, and integrity of the product and ensure the grease sealing performance and resistance to water erosion. The powder is used to adjust the consistency of the sealing grease, such as calcium carbonate. Base oil, such as mineral oil, vegetable oil, and synthetic oil, governs the fluidity and lubricity of sealing grease.

**Figure 1:**
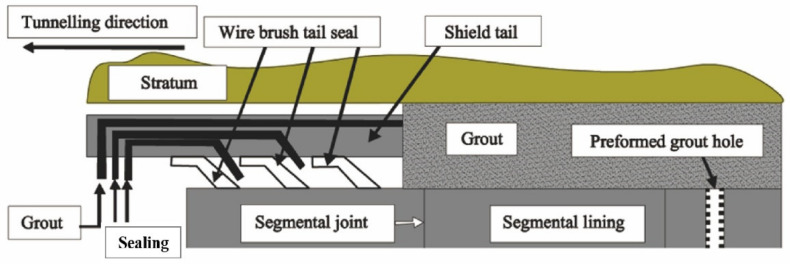
Schematic drawing of a shield tail seal system.

The quality of the shield tail seal has a decisive effect on the effective driving progress of the shield tunnel. The shield tail seal system consists of a steel wire brush, protective plate, and sealing grease, as shown in Figure 1. The sealing grease is pumped to the cavity using a pressure pump in the process of shield driving in the tunnel. Sealing grease is painted on the tunnel walls with the wire brush to form a sealing ring that is used to resist the grouting pressure and keep the slurry and soil out of the shield shell. Once the sealing grease fails, it will cause leakage in the shield tail and ground subsidence or even collapse of the tunnel [2].

The performance of sealing grease is mainly indicated by its pumpability and the pressure resistance of sealing. Research showed that the higher the fiber content, the lower the pumpability, and the better the sealing effect [3]. Therefore, fiber content is important for good sealing and pumping performance. In order to avoid sealing grease blockage in the pumping process, the fiber content has to ensure the highest pumpability, apart from a good sealing performance. In conclusion, the optimum fiber content is the content that just meets the sealing requirements. However, the optimum fiber content is varied in different studies and patents. It is often determined with experiment tests, which lack a theoretical basis. According to ASTM D1092, the pumpability is the grease volume flowing through a 25.4 mm hole per minute in conditions of 25 °C and the pressure of 1.0 MPa [4]. The pressure-resistant sealing performance of the grease is usually tested using laboratory experiments. A column with a sealed upper surface is used to mimic the shell and lining. A sieve with a specific opening size is laid on the bottom surface of the column to mimic the steel wire brush shown in Figure 1. After the sealing grease sample is evenly and tightly brushed on the sieve, pressure is applied to extrude the powder, leaving a dense “fiber cake” on the sieve. The sealing state would be reached while no powder is extruded from the sieve and no porosity occurred on the cross-section of the sealing grease sample [4,5].

Due to the high fluidity and large deformation nature of sealing grease, it is time-consuming and costly to determine the grease design via experiments. The development of a robust numerical method to optimize the properties of sealing grease has become a scientific challenge of main interest. The commonly used numerical methods include finite element analysis (FEA) and the discrete element method (DEM). In general, it is hard to develop FEA mesh when there are great discontinuities, which may result in the obstacle of meshing, ill-conditioned equations, or singular equations, leading to inaccurate results [5,6,7,8]. DEM is a more appropriate tool for solving mechanical problems involving discontinuities and large deformation. As a composite constituted of multiple components, sealing grease presents a heterogeneous nature and apparent discontinuity, which can reduce porosity in the process of pressure-resistant sealing tests. Fiber content is important for good sealing and pumping performance. Compared with the study only considering the effect of fiber length on polymer [9,10], it is more reasonable to take the fiber length diameter ratio as the variable. Therefore, the DEM model was used in this study to investigate the influence of different factors on the optimum fiber content.

DEM, originally developed by Cundall and Strack [11], is a very useful numerical tool for modeling the behavior of granular and particulate material. It has been adapted to study the fracture of brittle materials such as concrete, rocks, and composites [5,6]. In DEM, the materials are discretized by a great number of discrete elements interacting with each other. The discrete elements are spherical (three-dimensional), circular (two-dimensional), or polyhedral shapes. By setting the contact model and contact parameters between the discrete elements, materials with different properties can be obtained, such as flexible, elastic, bonding material, etc. In recent years, many scholars have studied the properties of fiber-reinforced composites by using discrete element method. Leclerc et al. [12] established a discrete element model to simulate jute fiber reinforced composites with the matrix of alumina and investigated the complex fracture phenomena occurring at very fine scales. Le et al. [13] established a discrete element model to study the interfacial debonding characteristics and the initiation and propagation of interfacial porosity in fiber-reinforced composites. Compared with experimental results, the simulation results fitted perfectly in terms of porosity propagation between fiber and matrix. Khattak et al. [14] researched the tensile response of carbon fiber-reinforced composite materials using DEM coupled with imaging techniques. The results obtained from DEM simulations were consistent with the experimental values, including elastic modulus and tensile strength of the molded carbon fiber-reinforced polymer composite. A DEM approach was established to investigate the impaction-sticking process during the initial deposition of fine particles on a single fiber by Yang et al. [15]. The model well predicted the formation of dendrites and the dynamic progression of deposit under different capture mechanisms [16]. Ganapathy et al. [17] established a DEM to study the synergetic effect of graphene and banyan fibers on the physical and mechanical characteristics of the epoxy thermoset.

In this article, DEM was used to simulate the sealing performance of sealing grease and investigate the influence of different viscosity grades and fiber’s aspect ratios on optimal fiber content, in which the viscosity grade was changed from 1 to 6, and the fiber’s aspect ratio was varied from 17 to 34. Meanwhile, the rationality of optimum fiber content obtained in the simulation was assessed by the maximum unbalanced force and the fiber area proportion of the models. Finally, the number of fiber spheres that represented the mass of fiber at the optimum fiber content was analyzed to provide a theoretical basis for practical production.

## 2. Discrete Element Modeling

### 2.1. Particle Flow Code

In this study, a model was established with Particle Flow Code (PFC), which can simulate the movement and interaction of multiple finite-sized particles [18,19]. The particles were rigid bodies with finite mass that moved independently in terms of translation and rotation. The interaction between the particles is determined by the internal force and moment at the contact interface between the pair-wise particles. The contact mechanical model works based on the particle contact law, in which the internal forces and moments were successively updated. It computes the time evolution of the particle interaction through DEM and derives the explicit dynamic solution to Newton’s laws of motion. The PFC model can mimic synthetic materials constituted with the assembly of rigid grains that interact at the contact interfaces, including both granular and cementitious materials.

**Figure 2:**
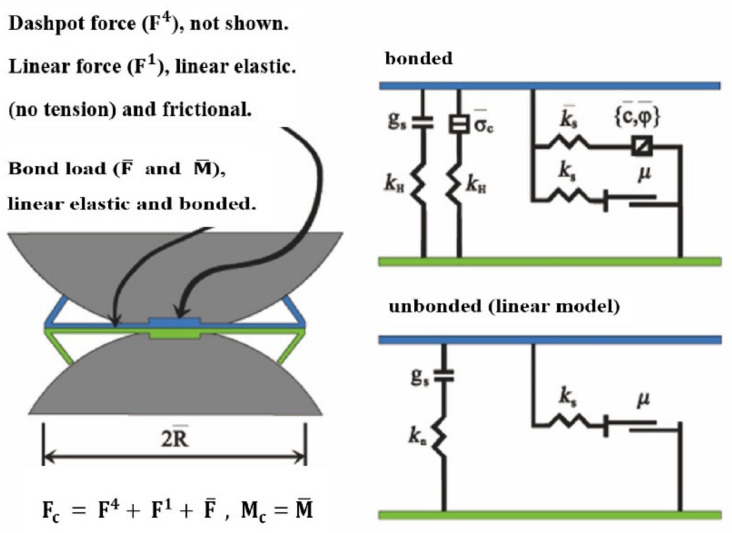
Illustration of the parallel bond model.

There are many contact models in PFC, such as the Hertz contact model, linear model, and linear contact bond model [20,21]. The parallel bond model was usually used to simulate cementitious materials [22,23], which can be used to simulate the mechanical behavior of two contacting spheres with a finite-sized piece of cement-like material deposited between them (similar to the epoxy cementing glass beads shown in Figure 2). The parallel-bonding effect was applied to the linear elements and established an elastic interaction between the elements. The parallel bond can transmit both force and moment between the spheres without precluding the relative slip [24].

A PFC model is composed of discrete elements (DEs) and the contact elements between DEs (contact model). In this study, DEs were used to model the sealing grease sample and the test device for sealing performance, and contact model parameters depended on the properties of the tackifier. In order to ensure the consistency between the parameters of the contact model and the properties of the actual sample, rheology tests were conducted to guide the selection of the contact model and model parameters. The predetermined parameters were then adopted in the subsequent models to investigate the effects of viscosity and fiber’s aspect ratio on the optimum fiber content.

### 2.2. Discrete Element—Geometric Model

The sealing grease model was composed of powder, fiber, and a tackifier (anchoring agent and base oil), and the testing device of sealing performance was composed of a pressure column and sieve.

**Figure 3:**
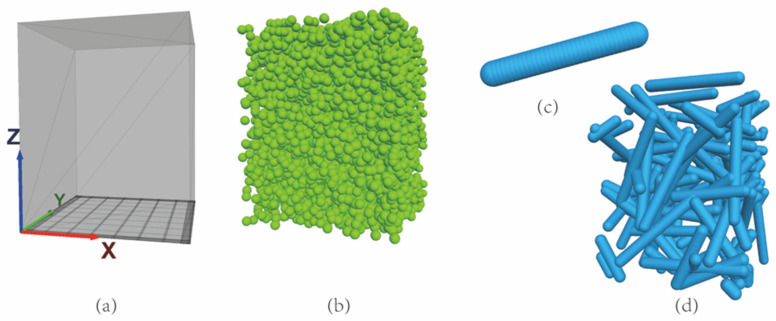
Snapshots of (**a**) the pressure column and sieve models, (**b**) powder model, (**c**) single fiber model, and (**d**) fiber model used in simulation.

- The pressure column was modeled with a 40 mm × 40 mm × 50 mm rectangular. The sieve was modeled using a series of regularly arranged triangular unit walls with the screen opening size of 2.4 mm × 2.85 mm and 5 mm × 5 mm for sealing tests and rheology tests, respectively, as shown in Figure 3a.
- A total of 5533 powder spheres were generated using the “ball distribute” command, with a porosity of 0.6 and a radius of 1 mm (Figure 3b) [25,26,27]. The selected size of the powder spheres was small enough to extrude from the sieve openings, although it cannot be the same size as the actual particles at micron order.
- The model of single fiber was created by a series of spheres with a radius of 1.5 mm and an embedded length of 0.5 mm (Figure 3c) [9]. It looked similar to a pearl necklace where the pearls geometrically interpenetrate with each other. A parallel contact model was adopted between the spheres, which can guarantee that the fiber does not suffer a compressive fracture during compression. The number of spheres for every single fiber depended on the fiber’s aspect ratio, while the fiber concentration determined the number of fibers. A series of fibers with a certain number and random direction were generated in the pressure column using a nested loop structure, as shown in Figure 3d. The physical parameters of the models for the sealing grease simulations are provided in Table 1.
- The tackifier acts as a bond between fiber spheres and powder spheres, which was simulated by a contact model rather than spheres. The parameters of its contact model were determined by the type of tackifier, which is significant for the properties of sealing grease samples.

### 2.3. Computational Procedure of Pressure Resistant Sealing

**Figure 4:**
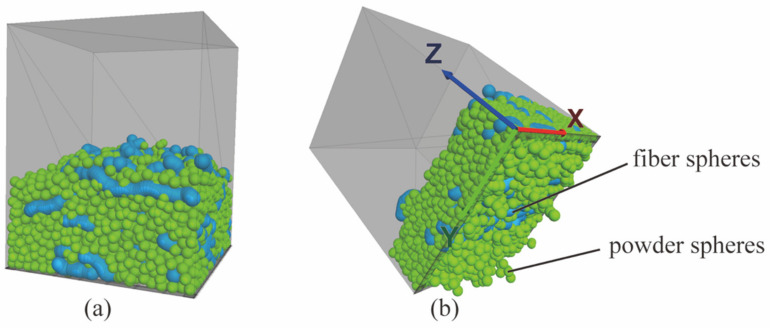
Snapshots of (**a**) the sealing grease arranged on the top of sieve evenly and tightly, and (**b**) the process of pressure resistant sealing test.

First of all, a pressure of 1000 Pa was applied to the sealing grease spheres along the negative *z*-axis. The simulation was calculated for 5000 steps to make spheres evenly and tightly arranged on the top of the sieve, as shown in Figure 4a. Secondly, the bottom wall of the pressure column was removed. A pressure of 3.5 MPa was applied on the sealing grease spheres along the negative *z*-axis to simulate the pressure-resistant sealing test (see Figure 4b). During the calculation process, the true time and the number of spheres extruded through the sieve (Number of Drops) were output. The function of applying low pressure (1000 Pa) is to compress the sealing grease into a relatively dense state. The pressure for the sealing performance test is generally 3.5 MPa [28].

### 2.4. The Criterion for Sealing State

Reaching the sealing state should satisfy the two conditions: firstly, there is no porosity on the cross-section of the sample. Secondly, the extrusion number of powder spheres is stable and not increasing.

### 2.5. Contact Element—Mechanical Modeling

In order to obtain reasonable parameters, six sets of sealing grease samples were prepared with the tackifier at different viscosities and measured for their rheological parameters by experiment and simulation. A good match between the simulation results and the experiment data would indicate that a reasonable contact model and proper parameters had been selected.

#### 2.5.1. Experiment of Rheology Test

The polyisobutylene with a molecular weight of 2400 g/mol (PB2400) and the polyisobutylene with a molecular weight of 1300 g/mol (PB1300) was used to prepare the sealing grease samples, of which the viscosity grades were defined according to the PB1300 to PB2400 mass ratio, as shown in Table 2. The time for 40 cm^3^ of the sealing grease sample being extruded from the hole under 1000 Pa pressure was measured using a melt flow rate instrument.

#### 2.5.2. Simulation of Rheology Test

In the DEM model, once the spheres were evenly and tightly arranged on the bottom of the pressure column, a pressure of 1000 Pa was applied to the sealing grease spheres along the negative *z*-axis. The volume of the sealing grease sample is 40 cm^3^, which is the same as the practical experiment. The total number of spheres extruded from the hole and the time of the sealing grease sample being extruded were recorded.

**Figure 5:**
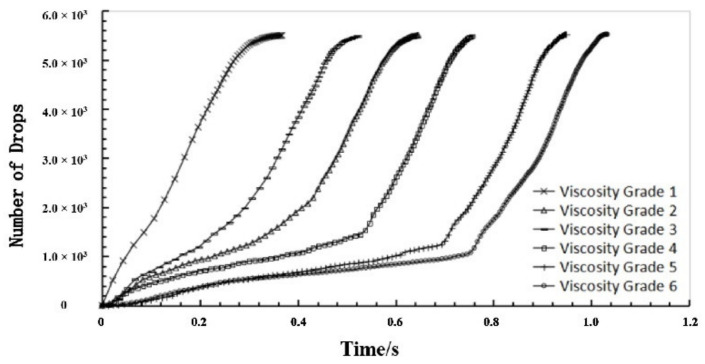
The curves between number of powder spheres squeezed from sieve and time under different viscosity grade.

**Figure 6:**
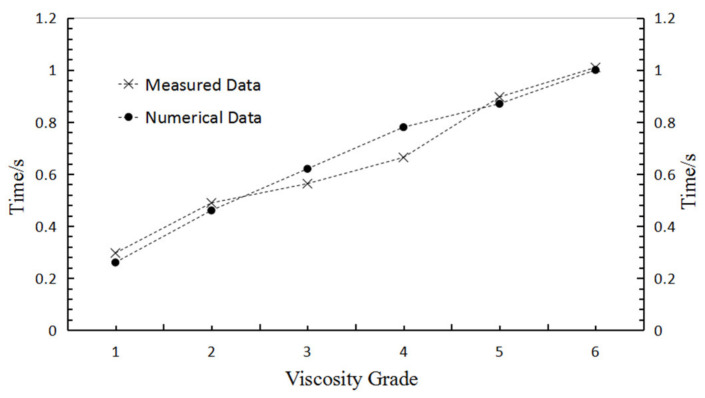
The contrast curves between measured data and numerical data.

The parameters of a parallel bond model include normal stiffness, friction force, normal critical damping ratio, tangential critical damping ratio, tensile strength, and cohesion. These parameters were adjusted using the variable-controlling approach. Specifically, one of these parameters changed continuously while other parameters remained the same. The viscosity grade of sealing grease would be influenced by this parameter in the condition of extrusion time of sealing grease, with the increase in parameter showing a regular increase. Results showed that only changing the critical damping ratio and tangential critical damping ratio can vary the viscosity of sealing grease. Therefore, the normal and tangential critical damping ratio were increased from 1 × 10^6^ to 3.5 × 10^6^ to adjust the viscosity of simulated grease, with the other parameters remaining constant, as shown in Table 2. The extrusion rate of the sealing grease samples at the six viscosity grades was plotted in Figure 5. When viscosity increases, the extrusion time of the sealing grease gradually increases, which indicates that the extrusion rate of sealing grease slowed down. The experiment measured data, and the numerical simulation data were compared in Figure 6. The measured data and numerical data are basically the same, illustrating a reasonable selection of the parameters for the numerical simulation model.

### 2.6. Parametric Study-Viscosity and Fiber Aspect Ratio

Six types of viscosity grade and five types of fiber aspect ratio were selected to study the effect of these factors on optimum fiber number. Determination of the optimum fiber number for different viscosity and fiber’s aspect ratio was studied by pressure-resistant sealing tests, which is the fiber number that just meets the criterion of reaching a sealing state. With unbalanced force or fiber area proportion, or the combination of these two, the rationality of optimum fiber number can be checked. Unbalanced force, generated in the iterative process, is the difference between the internal force and external force, which indicates the sealing effect of sealing grease. The smaller the unbalanced force and the greater would be the stability of the simulation model [29], which means that the displacement of powder spheres is smaller. In other words, the stability degree and convergence degree of the drops number are indicated by an unbalanced force. Fiber area proportion, the ratio between fiber area and total area of cross-section of “fiber cake”, is an indicator for the density of “fiber cake”. Thus, the number of porosities on the cross-section is indirectly indicated by the fiber area proportion. Specifically, optimum fiber numbers can be verified by drawing unbalanced force-fiber number curves and fiber area proportion-fiber number curves.

Finally, the relationship between the optimum fiber number and viscosity and that between the optimum fiber number and fiber’s aspect ratio were analyzed, which can be used to define the “Stable Zone” and the “Failure Zone” for sealing grease. The total number of fiber spheres indicates the mass of fiber. Therefore, the relationship curve of fiber’s aspect ratio against the total number of fiber spheres for the optimum fiber number was investigated.

## 3. Results and Discussion

### 3.1. The Effect of Viscosity Grade on Optimum Fiber Number

**Figure 7:**
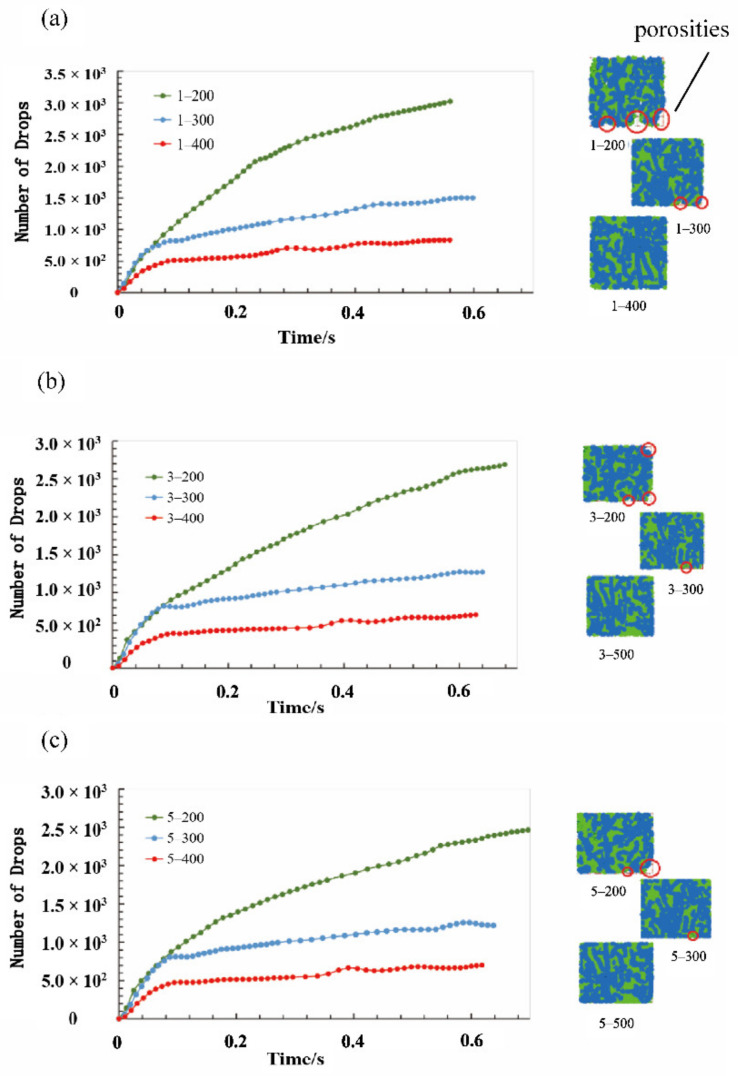
The development of drops number along the simulation and the cross section of the sealing grease models at (**a**) viscosity grade 1, (**b**) viscosity grade 3, and (**c**) viscosity grade 5.

Six viscosity grades of one to six were used to study the influence of viscosity on the optimum fiber number. The fiber’s aspect ratio was retained at 50 in the simulation process. During the computational procedure, the number of drops and real extrusion time were output every 500 steps. The computational procedure was interrupted when one of the following two situations occurred: the number of drops remained unchanged, or porosity appeared on sealing grease. Then the maximum unbalanced force was recorded. The snapshot of the cross-section 5 mm above the sieve at the end of computation was output. Finally, the number of drops was plotted against the real extrusion time. Combined with the porosity pattern of the cross-section at the end of the computation, the effect of the viscosity on the optimum fiber content was analyzed. It should be noted that the curves were denoted by the symbol of “viscosity grade-fiber number”, as shown in Figure 7. For example, the “1–200” symbol means the sample with the viscosity grade of one and fiber number of 200 used in the simulation model. The corresponding snapshots of the cross-sections were drawn beside the curves with the porosity marked using. The vacancy in the red circle in the Figure 7 refers to the porosities in the cross-sections of the model, indicating that the seal state has not been reached.

As shown in Figure 7a, when the fiber number is 200, the number of drops gradually increases without any convergence trend, and there was three obvious porosities on the cross-section. With the fiber number increasing to 300, the number of drops began to converge from 0.42 s and was stable around 1000. However, there was two tiny porosities. When the fiber number increased to 400, the increasing trend of the number of drops stopped from 0.39 s, which was stable at around 600. Meanwhile, there was no porosity on the cross-section. The pattern of the curves and porosity in the models illustrated that the sealing grease model could not reach a sealed state until the fiber number increased to 400 for viscosity grade 1. Above all, the fiber number just meeting the seal requirement should be between 300 and 400. In other words, the optimum fiber number for the sealing grease at the viscosity grade of one should be between 300 and 400.

**Figure 8:**
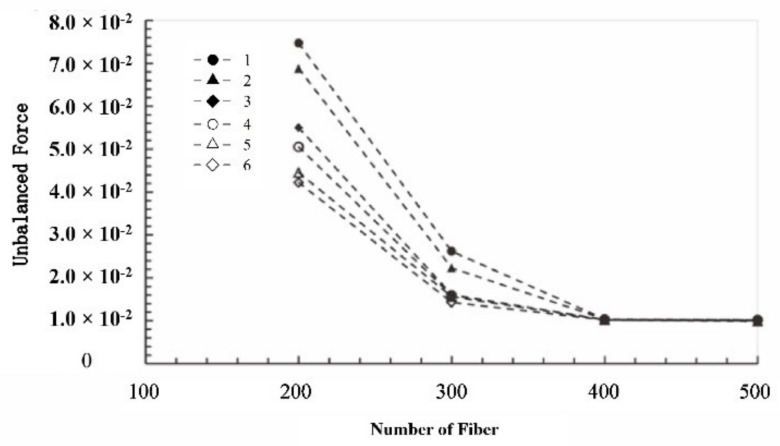
The relationship curves between unbalanced force and number of fibers for different viscosity grade (the symbols stand for the viscosity grades).

Similarly, the optimum fiber number of the other five viscosity grade was analyzed, which was the same as the fiber number of viscosity grade one. The simulation results of the models at viscosity grades three and five were shown in Figure 7 since the curve patterns of the models at different viscosity grades were consistent. Furthermore, the predicted optimum fiber number was assessed with the unbalanced force at the end of the computation. The unbalanced forces of the model with different fiber numbers and the six viscosity grades were plotted and compared. As shown in Figure 8, the unbalanced forces all decreased as the fiber number increased from 200 to 400 (200, 300, and 400). When the fiber number was higher than 400, the unbalanced force remained constant around 1.02 × 10^−2^. This indicated that the seal effect and seal state remain unchanged, and thus the fiber number of the model reaching the critical seal state should be around 400. Although the optimum fiber numbers for seal state were similar for different viscosity grades, the unbalanced force decreased with the increase in viscosity grade. This implied that the increase in viscosity could improve the sealing effect without changing the seal state. The higher the viscosity grade, the greater the flow resistance, which is consistent with the experimental results. Fibers build upon the sieve, reducing the flow cross-section of the seal grease. Therefore, an increase in fiber content and viscosity results in an increase in unbalanced forces.

### 3.2. The Effect of Fiber’s Aspect Ratio on Optimum Fiber Number

Based on the above simulation results, viscosity showed little influence on the optimum fiber number. However, the increase in viscosity can improve the sealing effect, apparently. Per conservativeness, the sealing grease at the smallest viscosity (viscosity grade 1) was selected in the subsequent simulation. Five aspect ratios of fiber: 17, 20, 24, 28, and 34, were used in the models to investigate the influence of fiber’s aspect ratio on the sealing performance and thus determine the optimum fiber number.

**Figure 9:**
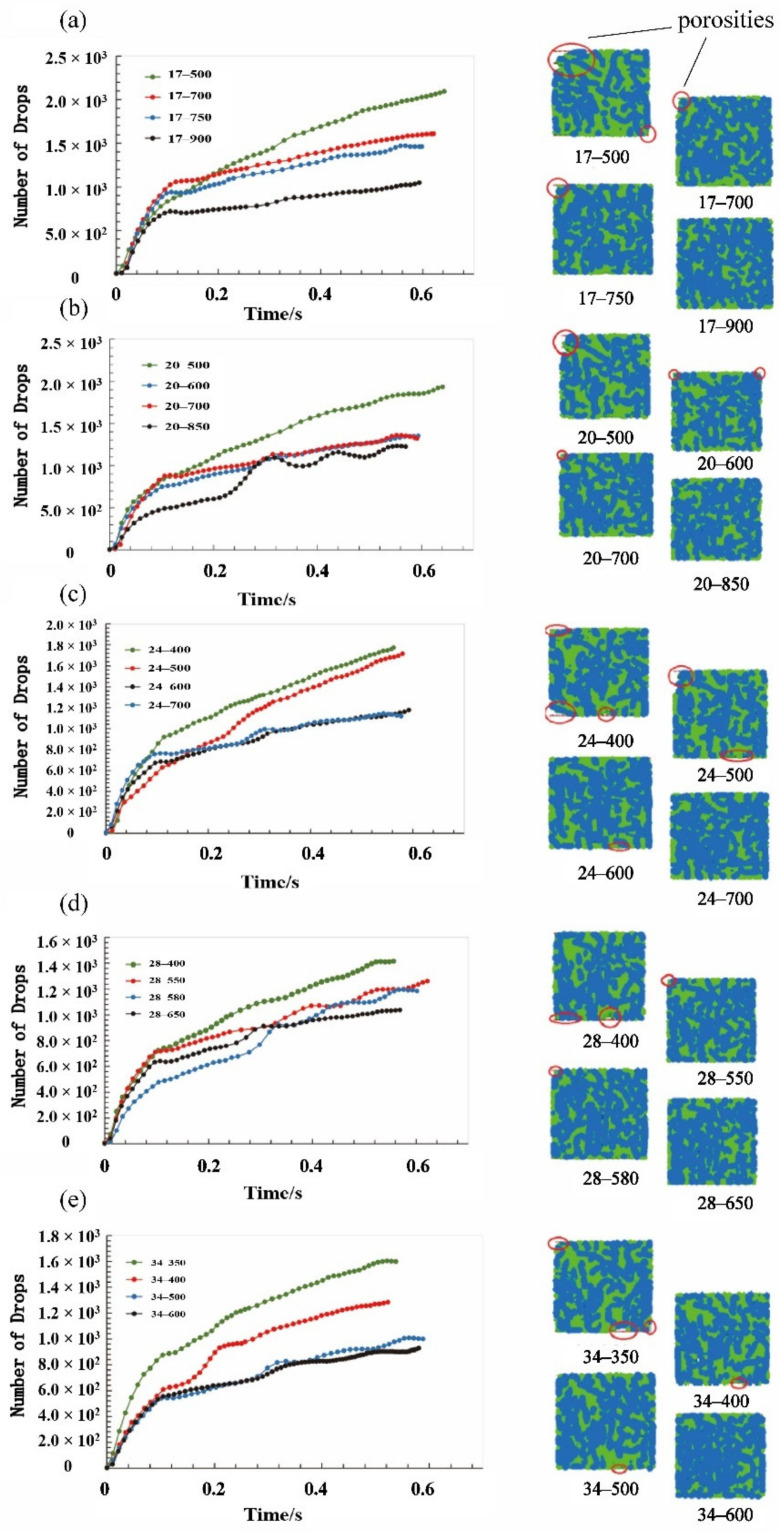
The development of drops number along the simulation and the cross section of the sealing grease models at different fiber’s aspect ratio, including (**a**) 17, (**b**) 20, (**c**) 24, (**d**) 28 and (**e**) 34.

The number of drops was plotted against the computation time in Figure 9 for the fiber’s aspect ratio varied from 17 to 34. For the model with the aspect ratio of 17, the number of drops gradually increased without a convergence trend when the fiber number was 500 and 700, as the curves show in Figure 9a. In addition, there were several porosities that appeared in the cross-sections of these two models, in which the seal state was not reached. Until the fiber number increased to 750, the number of drops began to converge from 0.48 s, and its value was around 1400. However, there was one tiny porosity on the top left corner of the cross-section, which indicated that the sealing grease sample with a fiber number of 750 did not reach the seal state. When the fiber number was increased to 900, the two criteria for reaching the sealing state were both fulfilled. Therefore, the optimum fiber number should be between 750 and 900. Similarly, as shown in Figure 9 and Table 3, the optimum fiber number for the other models with the aspect ratio of 20, 24, 28, and 34 should be between 700 and 850, 600 and 700, 500 and 600, 580 and 650, respectively.

**Figure 10:**
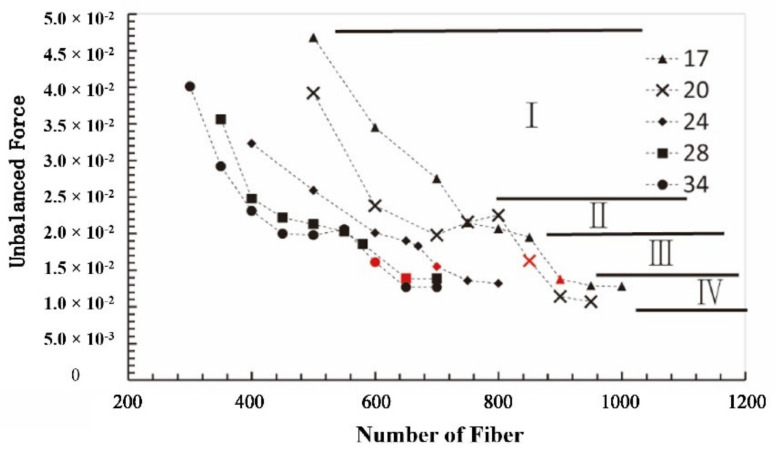
The variation curves of unbalanced force with fiber number increasing for different fiber aspect ratio, which can be divided into four stages: I, II (the first stable period), III, IV (the second stable period). The red symbol is the optimum fiber number determined hereinbefore.

Furthermore, the reasonability of the determined optimum fiber number was assessed by combination with unbalanced force and fiber area proportion. For different aspect ratios of fiber, the variation curve of unbalanced force with the fiber number increasing is shown in Figure 10, in which the red symbol is the optimum fiber number determined hereinbefore. The variation curves can be divided into four stages. For the fiber’s aspect ratio of 34, the unbalanced force gradually decreased to about 0.02, with the fiber number increasing from 260 to 440, which is the first stage. In the second stage, the unbalanced force remained 0.02 until the fiber number increased to 560, which was called the first stabilization period. However, the corresponding cross-section still had several tiny porosities, as shown in Figure 9e. Along with the fiber number increasing from 560 to 620, the unbalanced force decreased, and there was no porosity on the cross-section. It is the third stage, also called the critical seal period. Therefore, the optimum fiber number should be selected in this stage. In the fourth stage, with the increase in fiber number, the unbalanced force was around 0.012 without variation, and there was no gap on the cross-section, which implied that the sealing effect and seal state of the model were the same as in the third stage. Therefore, this stage is called the second stabilization period. Similarly, the other curves also can be divided into four stages, which are the same as fiber’s aspect ratio of 34.

**Figure 11:**
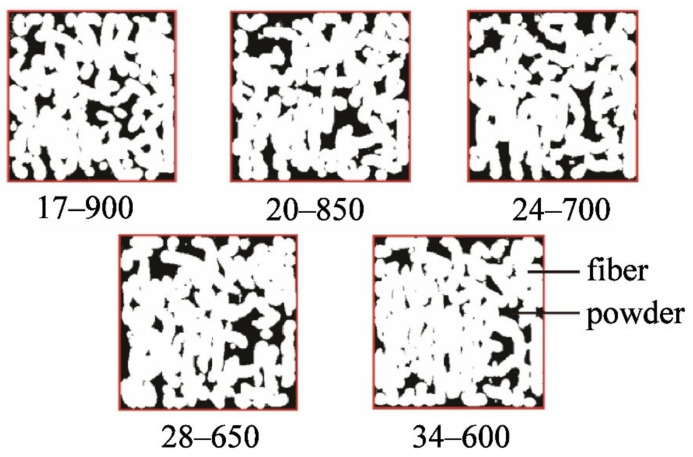
A series of cross section images used to analyze fiber area percentage at the optimum amount of fiber by Image Pro Plus image processing software (Image Pro Plus 6.0, Media Cybernetics, Bethesda, MD, USA).

Figure 11 presents a series of cross-sectional images used to analyze the fiber area proportion of “fiber cake” obtained with Image Pro Plus image processing software. Firstly, the entire cross-section surrounded by red lines was regarded as AOI (Area of Interest), and the total cross-sectional area was recorded as A_1_. Subsequently, the white region in the figure was regarded as AOI, and the corresponding area that is recorded as A_2_. Therefore, the proportion of the fiber area can be calculated by A_2_/A_1_. For each aspect ratio of fiber, the statistical fiber area proportion of “fiber cake” was summarized in Table 4.

**Figure 12:**
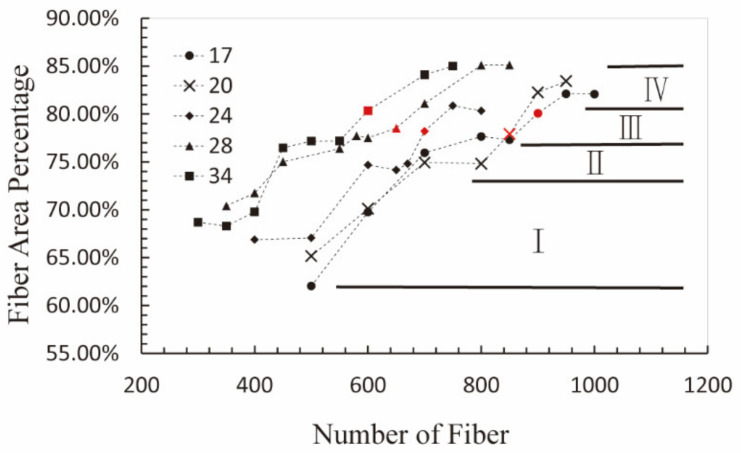
The variation curves of cross-section fiber area percentage with fiber number for different fiber aspect ratio, which can be divided into four stages: I, II (the first stable period), III, IV (the second stable period). The red symbol is the optimum fiber number determined hereinbefore.

The variation curves of fiber area proportion with fiber number increasing are shown in Figure 12. For the fiber’s aspect ratio of 34, this variation curve was divided into four stages. Firstly, as the fiber number increased to 440, fiber area proportion gradually increased to about 76%. Subsequently, the fiber area proportion was around 76% without variation, along with the fiber number increasing to 560, which was similar to the variation curve of unbalanced force in Figure 10. This illustrated that the sealing effect kept the same with the increase in fiber number. In the third stage, the fiber area proportion exceeded the critical value of 76% and reached a critical seal state, with the fiber number increasing to 680. Therefore, the optimum fiber number should be selected in this section. When the fiber area proportion increased to around 82%, the fiber area proportion stayed unchanged as the fiber number increased to 740, which was the reason that the sealing effect kept the same in the second stabilization period, as demonstrated in Figure 10. The other variation curves of fiber area proportion for the models with the fiber’s aspect ratio of 17, 20, 24, and 28 were similar to this curve.

Four stages can be divided for both the variation curves of unbalanced force and fiber area proportion, which include the sealing failure stage, first stabilization period, critical seal period, and second stabilization period. Furthermore, the demarcation lines of every two stages for these curves are practically identical. The changes of unbalanced force and fiber area proportion were synchronous, which indicates that the convergence degree of the number of drops was connected with porosity on the cross-section.

**Figure 13:**
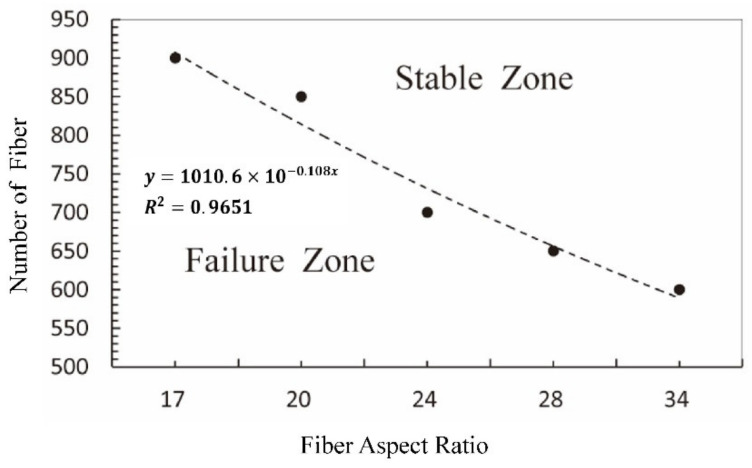
The fitting curve between the optimum amount of fiber and fiber aspect ratio.

After verifying the reasonability of optimum fiber number for different fiber aspect ratios, the optimum fiber amount at each fiber’s aspect ratio was plotted in Figure 13 with the dashed line as the fitting curve. The fitting curve can be expressed by the equation *y* = 1010.6 × 10^−0.108x^, and the relevant factor was 0.9651. The diagram showed that the optimum fiber number decreased gradually with the increase in the fiber aspect ratio. The sealing performance of the simulated grease was considered effective when the fiber number fell within the area above the fitting curve, denoted as “Stable Zone” in this study. On the other hand, the area below the curve was named “Failure Zone”, within which the sealing grease sample was considered to fail in sealing.

**Figure 14:**
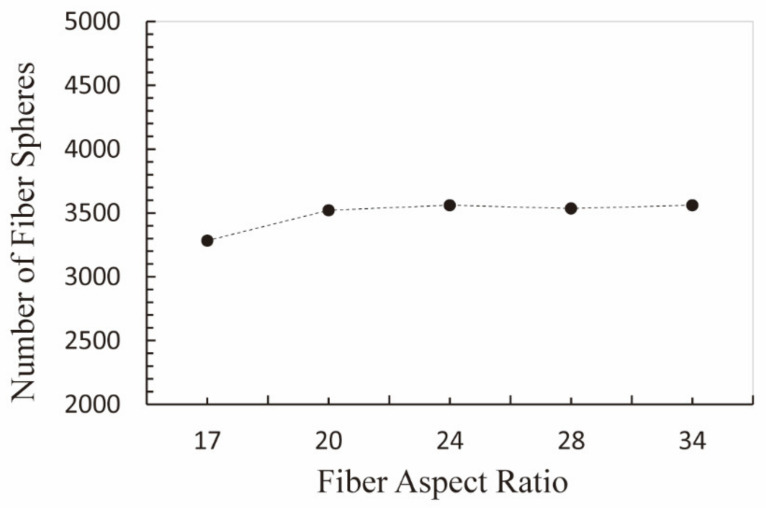
The variation of total fiber spheres number with fiber’s aspect ratio at the optimum fiber number.

Based on the above conclusion, the optimum fiber number decreased with the increase in the fiber’s aspect ratio. In other words, for the same grease volume, less fiber number would be required by the sealing grease with higher fiber aspect ratios to reach the same sealing state. The curve in Figure 14 presents the relationship between the fiber’s aspect ratio and the number of fiber spheres for the optimum fiber number. Each fiber sphere in the model was considered as one molecule of the fiber. The same quantity of fiber spheres means the same quantity of fiber molecules, and thus the same mass of fiber if sealing grease is made of the same material. The number of fiber spheres was unchanged and kept at about 3500 regardless of the optimum fiber number, which implied that the sealing grease sample made of the same fiber material and quality could reach the same seal state and seal effect, independent of the fiber’s aspect ratio.

## 4. Conclusions

The fiber content has a great influence on the performance of sealing grease. This study investigated the effect of the viscosity of a tackifier and the aspect ratio on the optimal fiber number using the discrete element method, which can provide a theoretical and statistical reference for sealing grease design. The conclusions are as follows:

(1) Viscosity grade had a significant impact on the pumpability of sealing grease, which was verified by experiment and DEM simulation of rheology tests. However, the viscosity of sealing grease showed little influence on the optimum fiber number. Noting that the increase in viscosity can improve the sealing effect, apparently.
(2) Compared with the influence of viscosity, the increase in fiber’s aspect ratio reduced the optimum fiber number, which was verified by the curves of unbalanced force and fiber area proportion of the DEM models. Furthermore, the variation curves of the unbalanced force and fiber area proportion were practically identical and can be divided into four stages. Although the optimum fiber number was dependent on fiber’s aspect ratio, the mass of fiber is related to the total number of fiber spheres. This study shows that the sealing grease sample made of the same fiber material and quality can reach the same seal state and seal effect.
(3) The DEM models and simulation results provided a solid and convenient tool to predict the quality of sealing grease. Many other parameters affecting the optimum fiber content and sealing performance, such as fiber type and fiber gradation, should be further considered in the DEM simulation for a more complete parametric study for guiding and improving the sealing grease design with numerical simulation.

## Figures and Tables

**Table 1:** Physical parameters of the models for the simulations.

Parameter	Dimension	Unit
Pressure column	4 × 4 × 5	cm
Screen opening of seal test	2.4 × 2.85	mm
Screen opening of rheology test	5 × 5	mm
Radius of powder	1	mm
Radius of fiber	1.5	mm
Length of fiber (34)	5	cm
Pressure (rheology test)	1000	Pa
Pressure (sample loading)	1000	Pa
Pressure (pressure resistant sealing test)	3.5	MPa

**Table 2:** The composition and contact model parameters of the sealing grease samples at different viscosity grades.

Experiment	PB1300:PB2400	5:0	4:1	3:2	2:3	1:4	0:5
Simulation	Normal stiffness (N/m)	1 × 10^5^	1 × 10^5^	1 × 10^5^	1 × 10^5^	1 × 10^5^	1 × 10^5^
	Friction force (N)	1 × 10^2^	1 × 10^2^	1 × 10^2^	1 × 10^2^	1 × 10^2^	1 × 10^2^
	Normal critical damping ratio	1 × 10^6^	1.5 × 10^6^	2 × 10^6^	2.5 × 10^6^	3 × 10^6^	3.5 × 10^6^
	Tangential critical damping ratio	1 × 10^6^	1.5 × 10^6^	2 × 10^6^	2.5 × 10^6^	3 × 10^6^	3.5 × 10^6^
	Tensile strength (Pa)	1 × 10^3^	1 × 10^3^	1 × 10^3^	1 × 10^3^	1 × 10^3^	1 × 10^3^
	Cohesion (Pa)	1 × 10^2^	1 × 10^2^	1 × 10^2^	1 × 10^2^	1 × 10^2^	1 × 10^2^
Viscosity Grade		1	2	3	4	5	6

**Table 3:** The drops number and the pore distribution at cross section at different fiber’s aspect ratio with different fiber number.

Fiber’s Aspect Ratio	Fiber Number	Number of Drops (Stability)	Pore Distribution at Cross Section
17	500	2078	One Large pore at up left corner and one tiny pore at bottom right corner
	700	1673	One tiny pore at up left corner
	750	1300	One tiny pore at up left corner
	900	993	No pores
20	500	1877	One Large pore at up left corner
	600	1256	Two tine pores at up left corner and up right corner
	700	1190	One tiny pore at up left corner
	850	967	No pores
24	400	1890	One large pore at bottom left corner and two tiny pores at up right corner
	500	1760	One large pore at up left corner and one tiny pore at bottom
	600	1077	One tiny pore at bottom
	700	923	No pores
28	400	1430	Two tiny pores at bottom
	550	1296	One tiny pore at up left corner
	580	1162	One tiny pore at up left coener
	650	960	No pores
34	350	1630	Two tiny pores at bottom right corner
	400	1295	One tiny pore at bottom
	500	921	One tiny pore at bottom
	600	867	No pores

**Table 4:** The statistical fiber area proportion of the optimum fiber number for each fiber’s aspect ratio.

Fiber’s Aspect Ratio	Cross-Sectional Area	Fiber Area	Proportion of Fiber Area
17	52,269	81,859	80.08%
20	80,052	62,264	77.91%
24	62,300	49,035	78.71%
28	85,069	66,796	78.52%
34	70,754	56,851	80.35%

## Data Availability

Data available in a publicly accessible repository.
